# Wearable Heart Rate Variability Monitoring, Autonomic Dysfunction and Post-exertional Malaise in Long COVID: An Observational Study

**DOI:** 10.1007/s40279-026-02487-4

**Published:** 2026-07-25

**Authors:** Twan M. Ruijgt, Anouk Slaghekke, Anneke Ellens, Kasper W. Janssen, Rob C. I. Wüst

**Affiliations:** 1 Department of Human Movement Sciences, Faculty of Behavioural and Movement Sciences, Amsterdam Movement Sciences, Vrije Universiteit, Van der Boechorststraat 7, 1081 BT Amsterdam, The Netherlands; 2 Sports and Exercise Medicine Clinic, DeSportarts, Dublinstraat 2, 3541 CD Utrecht, The Netherlands

## Abstract

**Objectives:**

Patients with long coronavirus disease (COVID) experience disabling fatigue, autonomic dysfunction, reduced exercise capacity and post-exertional malaise (PEM). Heart rate variability (HRV) can evaluate autonomic function and monitor overexertion, potentially helping to mitigate PEM. This study aimed to use continuous multi-day HRV recordings to monitor overexertion and study autonomic function in long COVID.

**Method:**

Heart rate and HRV were continuously measured in 121 patients with long COVID (43 ± 11 years, 32% male) and 21 healthy controls (42 ± 13 years, 48% male), with daily life activities tracked in a logbook. Participants underwent a (sub)maximal cardiopulmonary exercise test to determine heart rate at the first ventilatory threshold (VT1) to study HRV responses to exercise at different intensities.

**Results and Discussion:**

HRV was lower in patients with long COVID compared with healthy controls during various daily activities and sleep (*p* = 0.027). Across all exercise intensities surrounding the VT1, HRV remained lower for 24 h in patients compared with controls (*p* = 0.010). Nighttime HRV decreased with intense exercise and longer durations in patients with long COVID (*p* = 0.018), indicative of exercise-induced diurnal disturbances of the autonomic nervous system in long COVID.

**Conclusion:**

Heart rate variability, assessed by wearables, suggests autonomic dysfunction in patients with long COVID. The delayed recovery of the sympathovagal balance after exercise close to and above VT1, suggests that the risk of PEM rises above VT1.

**Application:**

These results confirm the applicability of wearables to assess autonomic function and manage overexertion in patients with long COVID.

**Supplementary Information:**

The online version contains supplementary material available at https://doi.org/10.1007/s40279-026-02487-4.

## Key Points

**Table:** 

***What is already known on this topic***
Patients with long COVID often experience fatigue, autonomic dysfunction and post-exertional malaise (PEM). HRV can be used as a non-invasive tool to measure autonomic function and recovery. Some evidence suggests lower HRV in patients with long COVID, but multi-day measurements are scarce.
***What this study adds***
This study demonstrates that continuous HRV monitoring through wearables can effectively identify overexertion and autonomic dysfunction during daily activities in patients with long COVID. Patients with long COVID have lower heart rate variability during sleep, and HRV remained significantly lower for a longer period after moderate-to-heavy exercise, that is generally associated with the induction of post-exertional malaise.
***How this study might affect research, practice or policy***
This study supports the use of wearables for assessing autonomic function and overexertion in daily life, helping patients with long COVID in pacing daily activities to mitigate symptoms of post-exertional malaise. HRV tracking after exercise suggests that exercise near or above VT1 provides an elevated risk for PEM. Sports physicians and physiotherapists can incorporate HRV biofeedback measures into pacing advice to patients. Additional research is needed to further investigate the effect of such an intervention.

## Introduction

Long coronavirus disease (COVID) is associated with several symptoms, including severe fatigue, a lower exercise capacity and unrefreshing sleep. Other commonly reported manifestations include autonomic dysfunction, such as postural orthostatic tachycardia syndrome (POTS) and post-exertional malaise (PEM), which is the worsening of symptoms after physical or mental exertion above an unknown patient-dependent threshold [1–4]. Many patients experience PEM following physical or mental exertion during daily life activities, leading to temporary worsening of symptoms and reduced functional capacity [1, 5]. Autonomic dysfunction is implicated in the pathophysiology, although the exact cause remains unknown [6–8]. Suggested interventions for patients with long COVID involve heart rate pacing and keeping exercise intensity below the risk zone for PEM, which is not precisely known. Also, triggers for PEM induction differ between patients [6, 9]. Since PEM is patient-dependent, a personalised approach in prescribing exercise and monitoring overexertion and recovery during daily activities is needed to improve patient well-being [5].

Heart rate variability (HRV) reflects the variation in time between consecutive heartbeats and provides a non-invasive measure of autonomic function [8, 10–12]. It represents the dynamic modulation of heart rate by the sympathetic and parasympathetic branches of the autonomic nervous system. A commonly used time-domain measure is the root mean square of successive differences between RR-intervals (RMSSD), which primarily reflects short-term, vagally mediated fluctuations in heart rate [13, 14]. While rMSSD is sometimes discussed in the context of sympathovagal balance, it most directly reflects parasympathetic modulation [12, 13]. Higher HRV is generally associated with greater autonomic flexibility and better recovery, although substantial inter-individual variability exists, partly related to age and sex [15–17]. Lower HRV is linked to reduced parasympathetic activity and increased physiological stress, making it a useful marker for autonomic function and recovery [18].

Patients with long COVID show mixed findings regarding heart rate variability (HRV). Some studies report lower HRV at rest or during sleep [8, 19], while others report higher HRV [20, 21], highlighting inconsistent findings across the literature. A recent systematic review confirmed that lower HRV is often reported in long COVID, but highlighted poor methodological quality, including some studies using very short measurement durations of only 1 min and lack of assessment in relation to specific daily life activities or sleep [22]*.* While these findings suggest autonomic dysfunction, characterised by diminished parasympathetic activity [8, 19], more studies are needed to study HRV in patients with long COVID.

Post-exertional malaise is a prominent feature in long COVID and is also commonly observed in patients with myalgic encephalomyelitis/chronic fatigue syndrome (ME/CFS) [23]. After exercise, HRV in patients with ME/CFS remained lower, while HRV increased in healthy controls [23], illustrating how autonomic dysfunction may relate to PEM. While these findings may provide relevant physiological context, the mechanisms underlying PEM in long COVID are unknown. Accordingly, the exact role of HRV in the pathophysiology of PEM in long COVID remains unclear.

Exercise intensity and duration significantly modulate the responses of the (para)sympathetic autonomic nervous system during exercise [24]. The parasympathetic nervous system is typically more active at rest and during low-intensity exercise, while sympathetic activity increases during high-intensity or prolonged exercise near or above VT1 [25]. How heart rate variability responds to acute exercise, and what the recovery responses after exercise are in patients with long COVID is, however, currently unknown.

Gaining a better insight into HRV responses during sleep, daily life activities and the recovery after moderate-to-heavy exercise will not only provide more fundamental insights into the pathophysiology of PEM and long COVID but also help patients with structuring daily activities and prevent overexertion and PEM. Therefore, this study aimed to investigate HRV over multiple days, during sleep, daily life activities and recovery after exercise near or above VT1 in patients with long COVID.

## Methods

### Participants

This retrospective study included patients with long COVID and healthy controls referred to a sports and exercise medicine clinic (DeSportarts, Utrecht, the Netherlands) between 1 January and 11 September 2024, who met the inclusion and exclusion criteria. Inclusion criteria for patients were (1) minimal one SaRS-CoV-2 infection, as diagnosed by a general practitioner and (2) being treated by a general practitioner for long COVID-related symptoms, including severe fatigue, brain fog, sleep disturbances and particularly post-exertional malaise. Control participants could have had a SaRS-CoV-2 infection but did not have long COVID symptoms. Exclusion criteria for the control group were chronic illnesses and related medication use or being an elite athlete.

Patients included in this retrospective study were receiving routine, medically specialised movement care at the sports and exercise medicine clinic (DeSportarts, Utrecht, the Netherlands). Current optimal treatment to avoid PEM in patients with long COVID includes avoiding overexertion and, as such, patients with long COVID were instructed, as part of routine clinical care, to avoid exercise above the heart rate at the first ventilatory threshold (VT1), using commercially available devices such as Garmin or Fitbit monitors to track heart rate. It is important to emphasize that pacing is a broader concept that encompasses more than heart rate limits; it also includes activity planning, scheduled rest and prioritization. In this study, we focused specifically on heart rate-based pacing as one component of these broader pacing strategies, with the aim of strictly controlling exercise intensity. Heart rate pacing was not advised in the control group.

The medical ethical committee of the Amsterdam UMC waived ethical oversight (2025.0092) in accordance with local legislation, as the heart rate variability (HRV) monitoring was part of standard care in a sports medical practice with extensive experience using HRV in patients and top athletes. The study design, involving the use of HRV devices for 3–7 days, was considered part of routine clinical practice. The study was deemed not to fall under the scope of the Dutch Research Involving Human Subjects Act by the Medical Ethics Committee of Amsterdam UMC. Local approval was obtained from DeSportarts Utrecht. All data presented here were derived from individuals who consented to provide their data for research purposes on the Firstbeat application. Before data analysis, participants were asked again for explicit consent to anonymous data analysis for the purposes of this research. No identifiable personal information was used during the analysis phase, ensuring participants’ privacy in accordance with ethical and regulatory standards for minimal-risk research.

### Patient and Public Involvement

Patients were not directly involved in the recruitment or conduct of the study, but their input was included during the development phase to refine the study design and ensure relevance to patient experiences and priorities. We held informal meetings with patients and their representatives and consulted them on the usability and practical challenges of heart rate-guided pacing, as well as the use of smartwatches for heart rate variability measurements and pacing strategies. We integrated their experiences into the interpretation of our data, and practical applicability of our results for real patients.

### Determination of Exercise Intensity Domains and Patient Severity

There is currently no scientific consensus about PEM induction, but anecdotal, clinical evidence suggests that the first ventilatory threshold (VT1) might provide some indications of such a risk zone [26]. VT1 reflects the point where the body begins to accumulate low levels of lactate in the blood, and marks the limit between aerobic and anaerobic exercise.

The first ventilatory threshold (VT1) was determined in a submaximal exercise test on a bike, 3–6 weeks prior to the HRV assessment. Owing to the retrospective design, the timing of the CPET relative to HRV assessment was not standardized. Termination criteria were pre-determined in consultation with the participant. If serious PEM symptoms were anticipated, the test was terminated after reaching the first ventilatory threshold (VT1), which was the case for most patients with long COVID. Otherwise, the test continued until the second ventilatory threshold (VT2) or maximal exhaustion.

The exercise test began with 2 min of rest on the ergometer followed by a 3-min baseline cycling period, followed by a ramped, linear increase in power output at a rate of 10–20 W/min in patients, 30W/min for healthy women and 40W/min for healthy men. Participants were instructed to maintain their cadence above 60 revolutions/min. Throughout the test, electrocardiogram (ECG), heart rate, blood pressure, O_2_ saturation levels and Borg exertion levels were monitored. Pulmonary gas exchange and ventilation were measured on a breath-by-breath basis (Cosmed Quark CPET; Cosmed, Rome, Italy), and displayed during the test. VT1 was determined using the validated *V*-slope and ventilatory equivalent methods ($\dot{V}$_E_/$\dot{V}$O_2_) and was assessed by two experts [27]. Heart rate at the first ventilatory threshold was defined as the start of the patient-specific PEM high risk zone for pacing strategies.

Exploratory post hoc analyses allowed us to classify patients with long COVID based on daily life impairments into mild and moderately impaired patients. We reasoned that approximately 15 ml O_2_/kg/min is needed for daily life activities [28], and that patients with a VT1 below this threshold may struggle more with daily tasks, as these would already bring them into the PEM risk zone. Patients with VT1 values ≥ 15 ml O_2_/kg/min were classified as mildly impaired, and patients with VT1 values < 15 ml O_2_/kg/min were classified as moderately impaired.

### Heart Rate Variability Assessments and Data Analysis

**Fig. 1:**
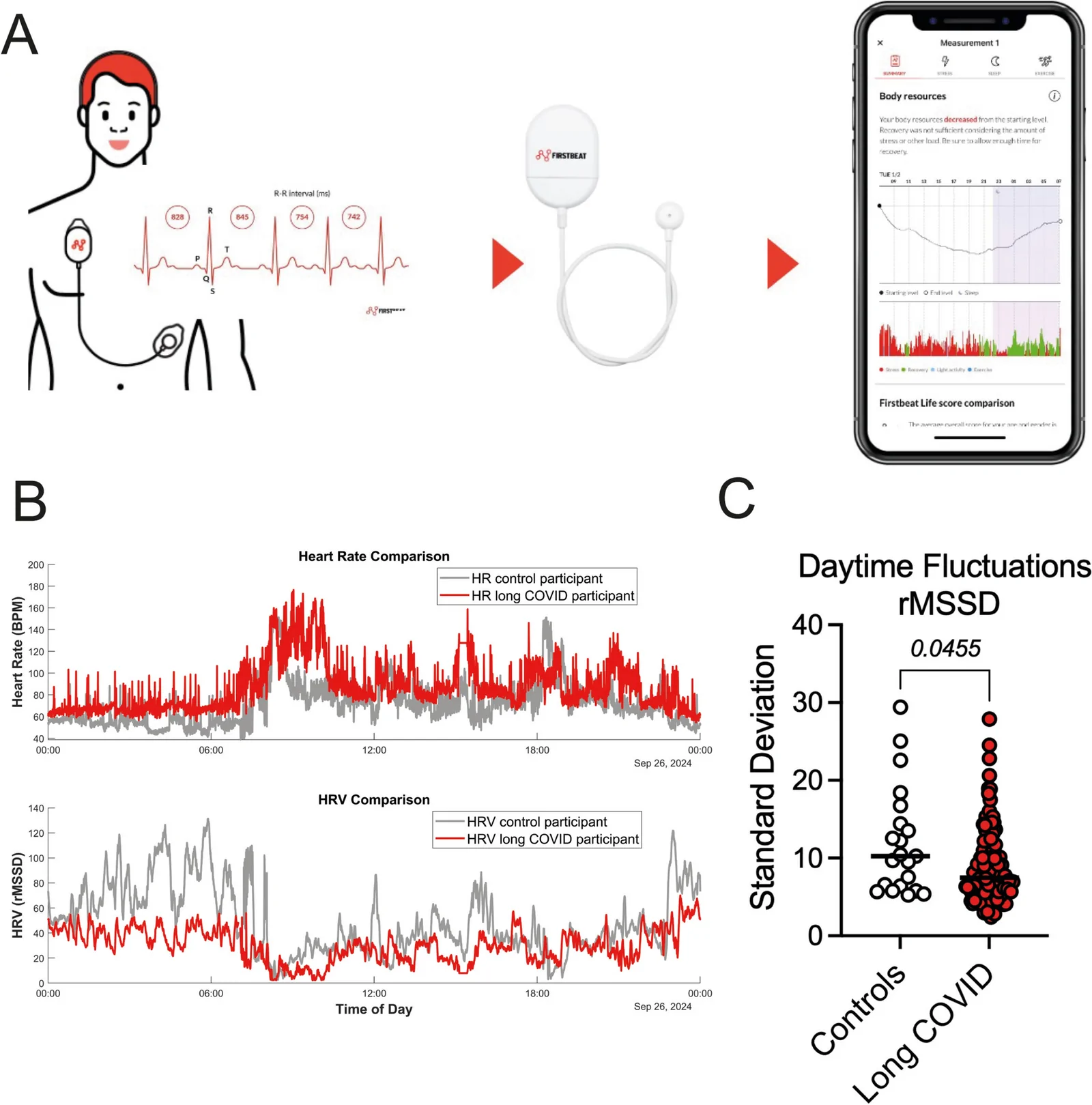
**A** Schematic overview of the measurement. The Firstbeat Bodyguard 3 device is attached beneath the right collarbone and left chest area. RR-intervals are recorded and stored in a digital database, and results can be viewed in an app. Data can be extracted for research purposes. **B** Typical example of heart rate and heart rate variability (HRV) recordings during one day for one healthy control and one patient with long COVID, with identical age, sex and BMI. HRV was calculated as the root mean square of successive differences between RR-intervals (RMSSD). When heart rate increased, HRV typically decreased. Healthy controls typically had more daytime fluctuations in HRV RMSSD compared with patients with long COVID. **C** The daily variability in HRV RMSSD, calculated as the average standard deviation over the day, was lower in patients than in healthy controls

All participants were instructed to wear a Bodyguard 3 device (Firstbeat, Finland), a clinical grade *ECG*-based HRV wearable, to measure heart rate variability (HRV). The device was attached using electrodes and placed beneath the right collarbone and below the left chest (CardinalHealth, Kendall, 57 × 34 mm; Fig. 1A). The Bodyguard uses a 1 channel ECG and measures RR-intervals with 1-ms intervals. Participants were instructed to wear the device for 5–7 consecutive days, excluding water contact (shower, bath etc.). Collected data were uploaded anonymously into the Firstbeat cloud. However, some participants removed the device earlier owing to personal reasons, such as vacations or skin irritation from the electrodes, resulting in an actual wear time of 3–7 days. Participants documented their daily activities in a logbook using the Firstbeat Life app. Retrospectively, these activities were further analysed and classified into strict domains: sleep, eating, travel, relaxation, work, walking, exercise, household chores, reading, relaxing exercises, getting ready and watching TV. Many of these categories were already available as standard options in the Firstbeat Life app, but additional refinement allowed for a more precise characterization of participants’ daily behaviours. HRV RMSSD, averaged over a 5-min periods by the Firstbeat software, was used for the analysis of daily life activities. HRV data for each category was averaged per participant across all measurement days to provide a personal, averaged HRV per daily life activity. This approach was chosen to allow a direct link between logbook recorded activities and HRV values calculated by Firstbeat, making it readily applicable in real-world settings. We calculated the daily variability in HRV as the standard deviation per participant for the different measurement days, using data collected between waking time and 20:00 in the evening.

For the HRV during recovery after exercise analysis, data analysis was conducted using Matlab R2023b. RR-intervals were filtered with a slightly adjusted version of HRVTool (v.1.07, https://marcusvollmer.github.io/HRV/). In short, a threshold-based beat correction algorithm was applied, using a 0.20 s threshold corresponding to the ‘medium-strong’ filter of the Kubios software. RR-intervals deviating by more than 0.20 s from the local average (median of 30 surrounding RR-intervals) were corrected using cubic spline interpolation. This threshold was adjusted on the basis of mean heart rate over a 20-s period; i.e. a heart rate of 120 bpm resulted in a filter threshold of 0.1 s. HRV was calculated using the root mean square of successive differences (RMSSD) from 300 RR-intervals. Heart rate was averaged over 20-s periods. A typical figure of HRV and HR throughout the day in both groups is shown in Fig. 1B.

#### Heart Rate Variability During Recovery After Exercise

Exercise intensities were categorised into three levels: mild (80% ≥ HR < 90% of VT1), moderate (90% ≥ HR < 100% of VT1) and intense (HR ≥ VT1). For every hour after exercise completion (up to 24 h), a 15-min average HRV was computed. Exercise was defined as maintaining HR above the predefined intensity for ≥ 20 min, and this was often accompanied by a comment in the diary. While it is unlikely that psychological factors such as stress, pain or fear would sustain for such periods, we do not exclude that cognitive exertion could have contributed in some instances [29]. Relative short segments of 15 min were used to capture transient post-exercise autonomic changes, which may have been masked by averaging over a full hour. The total time spent in each exercise level during the day was classified as short (< 20 min), medium (20–60 min) and long (> 60 min). Because not all healthy controls underwent a submaximal exercise test, VT1 was estimated using 75% of age-predicted maximal heart rate in 12 out of 21 participants (57%). In the subset of participants who completed the CPET (*N* = 9), VT1 occurred at a median of 0.80 of predicted maximal heart rate (interquartile range (IQR) 0.14), which falls within the limits of agreement and is consistent with the reference threshold of 0.75 [30].

Additionally, for each participant, the highest nighttime HRV across all measurement nights was used as a reference value. Similar to the concept of resting heart rate, we reasoned that the highest HRV value is the best value that participants could reach during the measurement period. We do not know exactly what activities were performed during the day and how this would impact HRV during the following night in patients with long COVID and, as such, average of baseline HRV was not used. The percentile difference for each night relative to this reference value was then calculated to evaluate the impact of exercise-induced stress on nighttime HRV.

### Statistical Analysis

Statistical analysis was performed using R (version 4.4.1). Descriptive data were reported as mean ± standard deviation, unless noted otherwise. Normality was checked using Shapiro–Wilk tests and visually inspected with Q–Q plots. Significance was accepted at *p* < 0.05.

Differences in daily-life HRV (RMSSD) were analysed using a non-parametric rank-based regression model (R-fit), with group (control versus long COVID) as the main factor, and age and sex included as covariates owing to their established associations with HRV (Supplementary Fig. 1). The normally distributed heart rate data were analysed using a linear model with the same covariates. Effect sizes were expressed as the raw mean differences (unstandardized coefficients) between both groups, derived from the respective models.

Differences in post-exercise HRV recovery were analysed using a rank-transformed mixed linear model to account for repeated measures, with severity group (control, mildly impaired, moderately impaired), time (recovery hours) and exercise intensity (80–90%, 90–100%, > 100% of heart rate at VT1) as fixed effects, and age and sex as covariates.

Exercise-induced alterations in nighttime HRV were analysed using a linear mixed-effects model on the mean relative change in nighttime HRV compared with each participant’s best night. Group, time spent per exercise intensity during the day and exercise intensity were included as fixed effects. Only absolute HRV measures were corrected for age and sex, while relative HRV changes were adjusted solely for repeated measures. When exercise was performed on multiple days, the participants' mean HRV across those days was used in the analysis. Post hoc testing of linear models was conducted using the *emmeans* package with Bonferroni adjustment for multiple comparisons.

## Results

This study included 121 patients with long COVID and 21 control individuals (patient characteristics in Table 1). Long COVID participants had, on average, a higher BMI compared with the age- and sex-matched healthy controls. Patients with long COVID had a lower power output and oxygen uptake consumption ($\dot{V}$O_2_) at VT1 compared with controls (*p* < 0.001). Patients with long COVID were classified as moderately impaired, with VT1 < 15 ml/kg/min (which is the oxygen consumption needed for daily life activities; *N* = 34, 24% male, 43 ± 11 years, $\dot{V}$O_2_: 13.1 ± 1.2 15 ml/kg/min) or mildly impaired (VT1 ≥ 15 15 ml/kg/min; *N* = 87, 36% male, 41 ± 10 years, $\dot{V}$O_2_: 21.6 ± 5.1 15 ml/kg/min).

**Table 1:** Participant characteristics

	Healthy control	Long COVID	*p* value
Number of participants	21	121	
*Daily life*			
Age (years)	42 ± 13	42 ± 11	*0.87*
Sex (male %)	10 (47.6%)	35 (31.0%)	*0.17*
Height (cm)	178 ± 9	174 ± 10	*0.14*
Weight (kg)	73.4 ± 13.0	77.6 ± 14.5	*0.22*
BMI (kg/m)	23.1 ± 3	25.7 ± 4.7	*0.02*
*Submaximal exercise test*			
Number of participants	9	121	
Age (years)	41 ± 15	42 ± 11	*0.74*
Sex (male)	4 (44.4%)	35 (31.0%)	*0.46*
BMI (kg/m)	23.9 ± 2.6	25.7 ± 4.7	*0.31*
Power output at VT1 (W)	183 ± 59	100 ± 44	< *0.001*
$\dot{V}$O_2_ at VT1 (ml/kg/min)	28.6 ± 6.1	19.2 ± 5.8	< *0.001*
RER at VT1	0.95 ± 0.04	0.95 ± 0.04	*0.74*

Data is shown as mean ± SD

### Adverse Effects from the CPET

Three patients from this cohort experienced self-reported PEM for more than 5 days after the submaximal CPET. More than 5 days was considered an adverse event, because of the prolonged negative impact on daily life. These patients were not excluded from the study owing to the significant time between the exercise test and the HRV measurement.

### HRV During Daily Life Activities

**Fig. 2:**
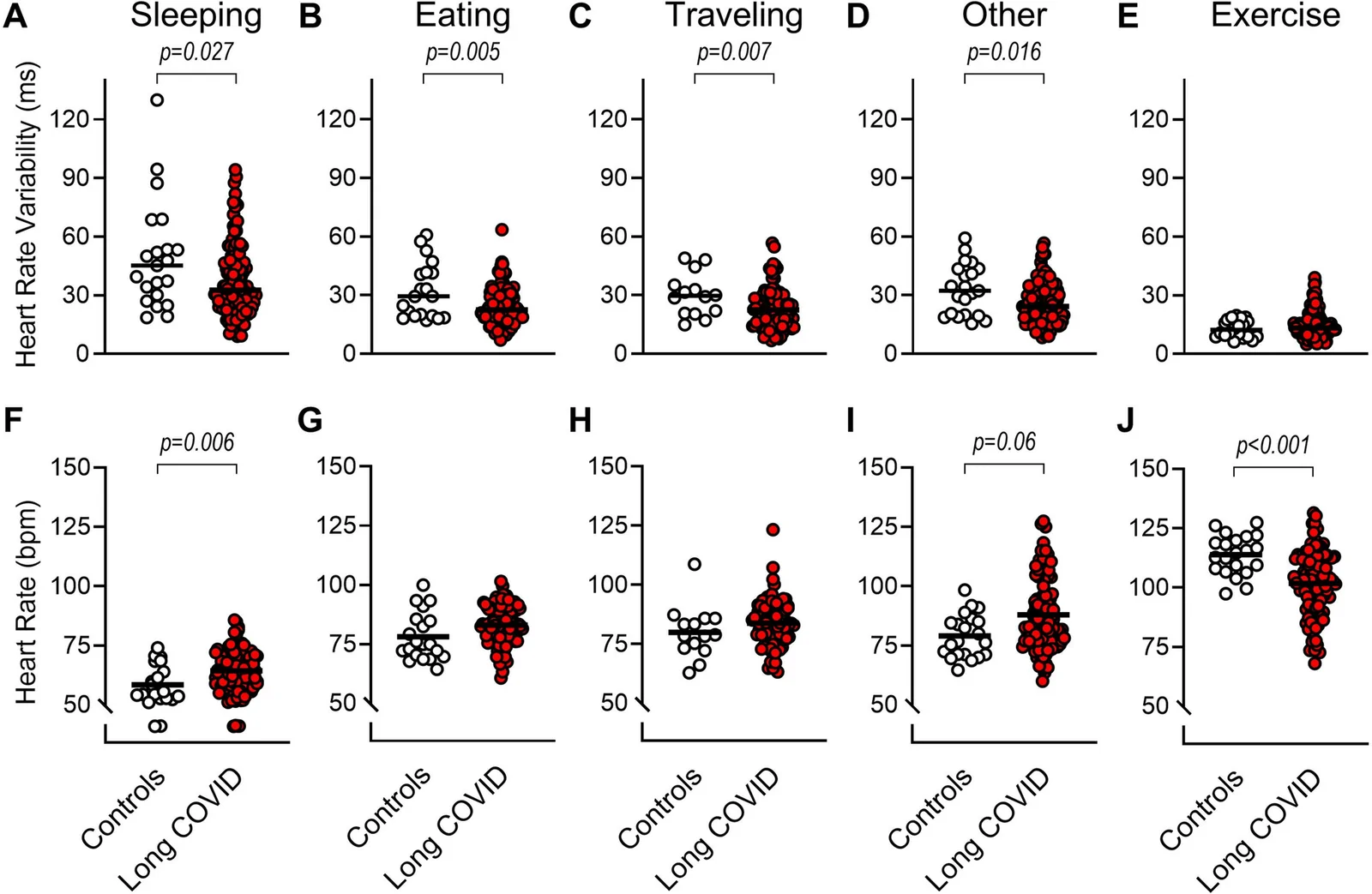
Heart rate variability and heart rate during daily life activities. HRV (**A–E**, measured as RMSSD) and heart rate (**F–J**, in bpm) are shown during different daily life activities: sleeping (**A, F**), eating (**B, G**), traveling (**C, H**), uncategorized activities (**D, I**) and exercise (**E, J**). HRV was significantly lower in patients with long COVID compared with healthy controls during sleep (*p* = 0.027), eating (*p* = 0.005), traveling (*p* = 0.007) and uncategorized activities (*p* = 0.016). No difference in HRV was observed during exercise (*p* = 0.25) (**E**), despite significantly higher heart rates in controls (*p* < 0.001, **J**). In patient with long COVID, heart rate was significantly higher during sleep (*p* = 0.006, **F**) and a tendency in uncategorized activities (*p* = 0.06, **I**), while no significant differences were observed during eating (**G**) or traveling (**H**). For the HRV data median values (horizontal bars) and individual data points are displayed

The daily variability in HRV RMSSD, calculated as the average standard deviation over the day, was lower in patients than in healthy controls. This indicates smaller fluctuations in HRV over the day in patients with long COVID. HRV RMSSD was lower in the long COVID group during sleep, eating, travelling and the uncategorised (and combined) activities (*p* = *0.005–0.027*; Fig. 2, Supplementary Table 1 and 2). Effect sizes and confidence intervals of the various daily life activities are mentioned in Table 2. Notably, 45% of reported activities were classified under this non-specific category, as these activities consisted of those that did not fall directly into one of the 12 predefined categories (e.g. shopping) or were a combination of two activities (e.g. eating in front of TV). A negative correlation between HR and HRV is shown in Supplementary Fig. 2. Heart rate was significantly higher during sleep and the uncategorized activities in patients with long COVID, but no such difference was observed during eating or traveling. The daily life activities with higher absolute heart rates and subsequently lower HRV values, such as exercise, showed no significant group differences in HRV.

**Table 2:** Unstandardized effect sizes, 95% confidence intervals and corresponding *p* values compared with healthy controls for the heart rate variability and the heart rate for the various daily life activities that were recorded by participants

Activity	Effect size (*Β*)	95% confidence interval	*p* value
*Heart rate variability (HRV)*			
Sleeping	− 8.82	[− 16.58, − 1.06]	0.027
Eating	− 6.19	[− 10.45, − 1.92]	0.005
Traveling	− 6.63	[− 11.38, − 1.88]	0.007
Other	− 5.82	[− 10.50, − 1.14]	0.016
Exercise	1.52	[− 1.07, 4.11]	0.25
*Heart Rate (HR)*			
Sleeping	5.21	[1.49, 8.92]	0.006
Eating	3.25	[− 1.51, 8.01]	0.18
Traveling	1.90	[− 3.28, 7.09]	0.47
Other	6.72	[− 0.23, 13.68]	0.06
Exercise	− 13.33	[− 19.13, − 7.53]	< 0.0001

See also Fig. 2

### Recovery of HRV After Exercise Cessation

**Fig. 3:**
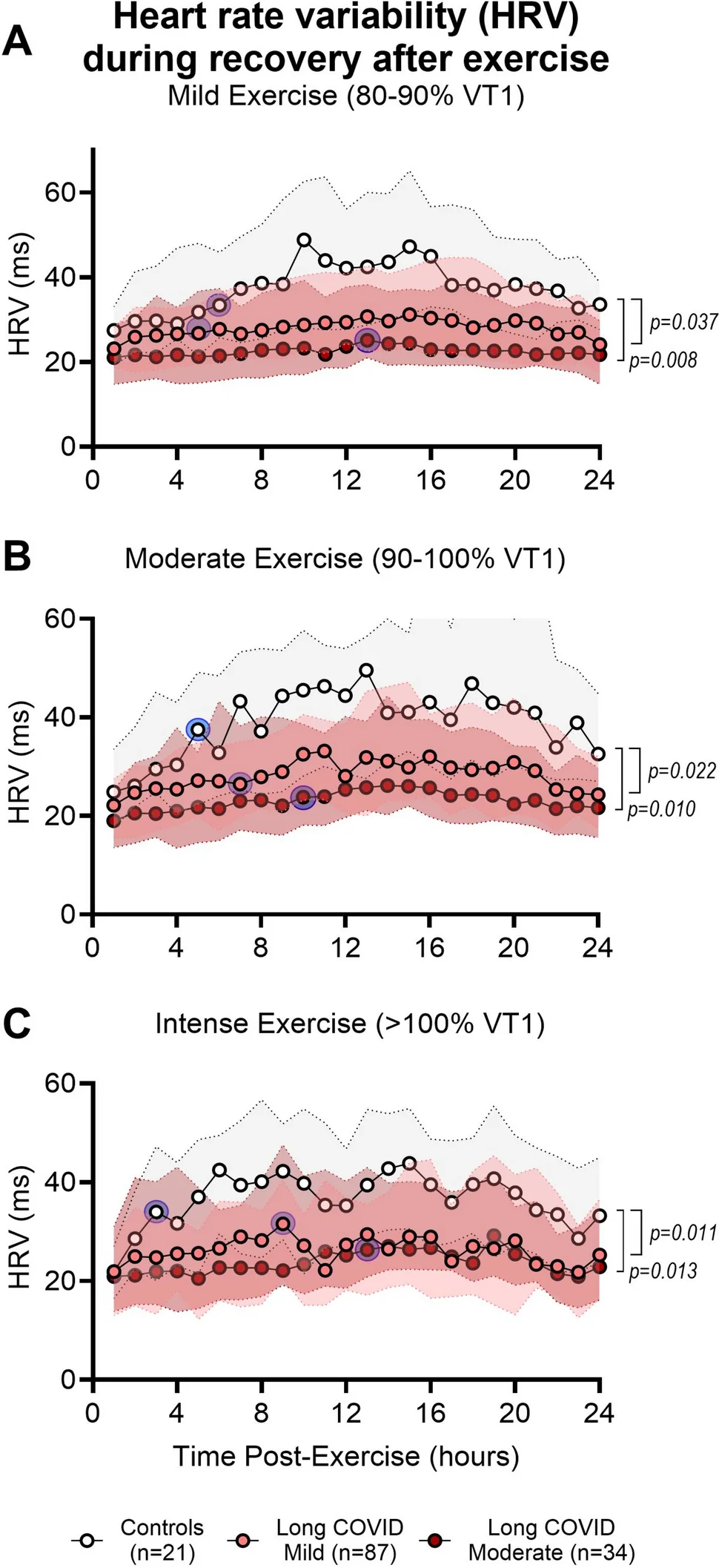
Recovery of heart rate variability (HRV) after exercise cessation. The time course of heart rate variability (HRV, as measured as RMSSD) for 24 h after cessation of exercise at mild (**A**: 80–90% VT1), moderate (**B**: 90–100% VT1) and intense (**C**: > 100% VT1) intensity. Healthy controls (white) had higher HRV compared with patients with mild (pink) and moderate (dark red) long COVID. Blue-shaded circles indicate the time points at which HRV values became significantly higher (*p* < 0.05) compared with the 1-h post-exercise baseline within each group. Data points represent the median HRV values for each group at each time point, with error bars indicating the interquartile range because data was not normally distributed

As heart rate pacing advice is the current optimal treatment for patients to avoid PEM, we first studied how many patients recorded heart rates above the VT1. In 43% of patients with long COVID, we recorded heart rates above the anaerobic threshold during daily life activities. Thus, the adherence to the heart rate pacing advice given after the CPET was 57%. We stratified exercise into three exercise categories: mild (80% ≥ HR < 90% of VT1), moderate (90% ≥ HR < 100% of VT1) or intense (HR ≥ value at VT1) exercise, and we studied the recovery of the heart rate variability after exercise cessation. For this analysis, we made a subdivision between mildly and moderately impaired patients on the basis of oxygen consumption at VT1. Figure 3 illustrates the time course of HRV during the recovery period after mild, moderate and intense exercise. During the hours following exercise, patients with long COVID showed significantly lower HRV values compared with healthy controls (*p* = 0.010), irrespective of exercise intensity, with no differences observed between mildly and moderately impaired patients. Compared with controls, HRV reduced the most after intense exercise in the mildly affected patients (*p* < 0.001), but not in the moderately impaired patients where HRV was consistently low. HRV increased relatively rapidly after exercise cessation in healthy controls (3–6 h post-exercise; Fig. 3 blue markers). This recovery-associated increase in HRV (which is independent of heart rate) was significantly faster in healthy controls compared with mildly and moderately impaired patients with long COVID (*p* = 0.002), indicating delayed autonomic recovery in patients with long COVID. While HRV increased relatively rapidly after mild intensity exercise in mildly affected patients (5–6 h), the HRV recovery was further delayed after exercise above VT1 (until 9 h). HRV did not increase, relative to the exercise itself, until 10–13 h in the moderately impaired patients at various exercise intensities above 80% of VT1. Similar results were obtained when sample size of both groups were the same (Supplementary Fig. 3 and Supplementary Table 3).

### Nighttime HRV Following Exercise

**Fig. 4:**
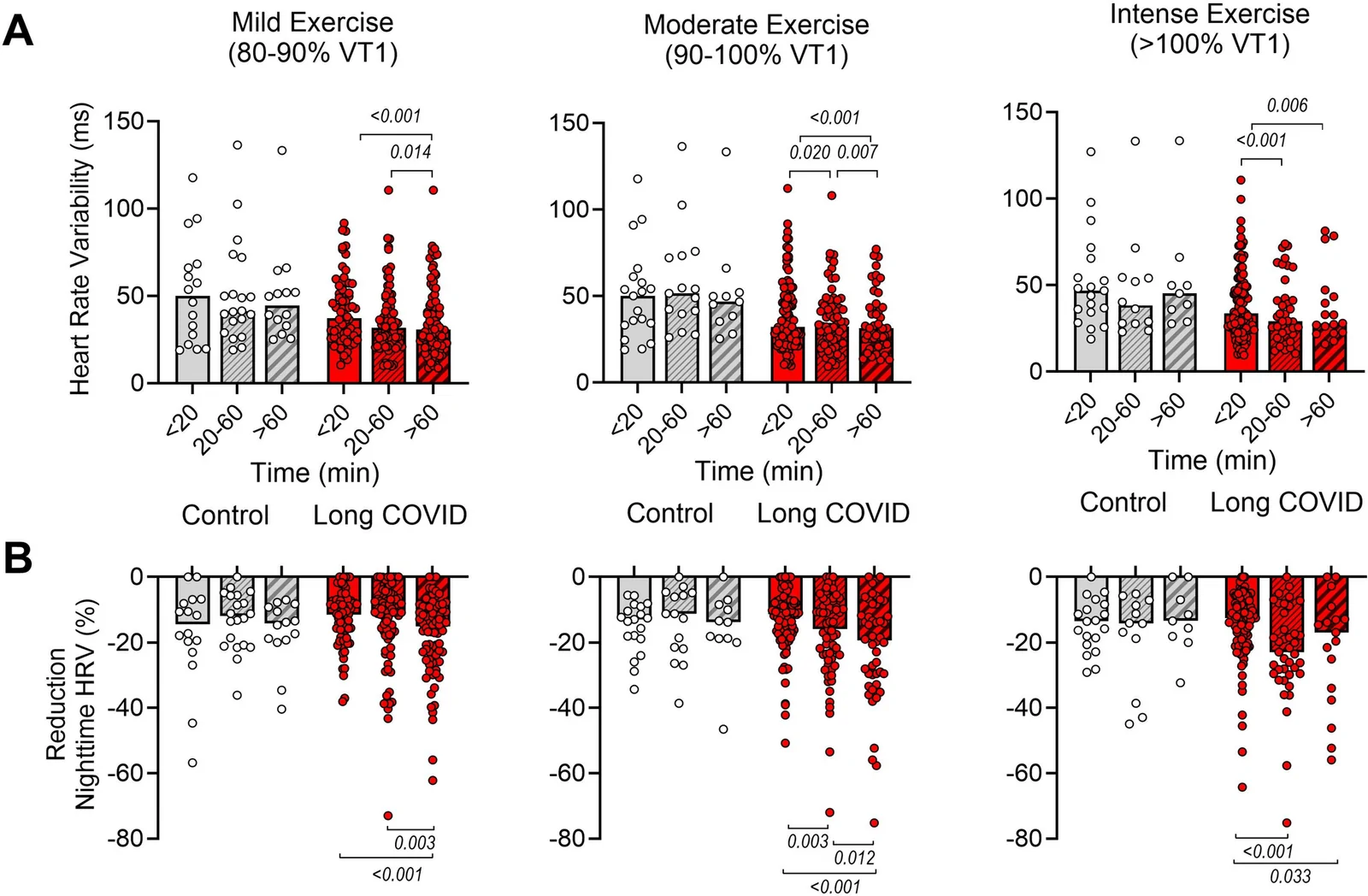
Heart rate variability during post-exercise night.** A** Heart rate variability (HRV) during the night following mild (80–90% of VT1), moderate (90–100% of VT1) and intense (> 100% of VT1) daytime exercise. HRV was lower, only in patients, following exercise with increasing duration (around the VT1) and exercise intensity. **B** The relative change (%) in nighttime HRV (measured as RMSSD) is shown following mild (80–90% of VT1), moderate (90–100% of VT1) and intense (> 100% of VT1) daytime exercise. Also the relative reduction was lower in patients, following exercise with increasing duration and exercise intensity. Exercise durations were divided into three categories: < 20, 20–60 and > 60 min. Bars represent group medians, and individual data points are shown

We reasoned that the delayed recovery time course in HRV in patients would eventually affect nighttime HRV. Indeed, nighttime HRV was consistently reduced following exercise, with differences across exercise durations and intensities (Fig. 4A). The nighttime HRV of healthy controls and patients reacted differently to the day-time exercise bout. In controls, nighttime HRV tended to be higher (*p* = 0.059), and was not significantly influenced by exercise duration or intensity. In patients however, increasing exercise duration and intensity both resulted in significantly lower absolute HRV values during the subsequent night. The interaction between exercise duration and intensity (*p* = 0.010) suggests that nighttime HRV depends on the combined effect of these two factors.

To further illustrate these effects, we analysed the relative reduction in nighttime HRV (%) compared with the reference value, defined as the highest nighttime HRV by each participant (Fig. 4B). In the long COVID group, both exercise intensity and duration significantly influenced the magnitude of nighttime HRV reduction. Specifically, engaging in more than 60 min of exercise, regardless of intensity, led to a greater reduction in nighttime HRV compared with performing < 20 min of exercise at the same intensity (*p* = 0.033). Additionally, a significant difference was observed between mild and intense exercise when performed for < 60 min, with intense exercise causing a greater reduction in nighttime HRV (*p* = 0.038). The significant interaction effect between time and intensity (*p* = 0.018) underscores the combined impact of both factors on nighttime HRV recovery, particularly in patients with long COVID. These results illustrate that exercise with increasing intensity and/or duration causes a delayed adjustment of HRV in patients with long COVID.

## Discussion

The aim of this real-life study was to use HRV during daily life activities to study autonomic function and the recovery responses to exercise in patients with long COVID. The adherence to the heart rate pacing advice was substantial, 57% of patients did not exceed their VT1 threshold during the HRV measurement. We observed that HRV was lower in patients with long COVID in multiple daily life activities compared with healthy controls and did not fluctuate as much during the day. HRV in patients with long COVID remained low during the recovery period after mild, moderate or intense exercise when compared with healthy controls. Lastly, long-lasting exercise around or above the VT1 resulted in a lower HRV during the subsequent night in patients with long COVID.

### Autonomic Dysfunction in Long COVID

Our findings of a lower HRV in patients with long COVID are consistent with earlier research [16–18]. The lower HRV during sleep and many daily life activities, and fluctuations during the day in patients compared with healthy controls, suggest that patients with long COVID may experience significant autonomic dysfunction, characterised by reduced parasympathetic activity and/or an increased sympathetic activity throughout a large proportion of their daily life, and during the night. The lower HRV observed during sleep likely limits the ability of the body to recover from daily stressors. This could partially relate to the unrefreshing sleep commonly reported in long COVID, although we did not specifically ask for sleep quality in the present study. The stronger age–HRV association in controls indeed likely reflects normal age-related autonomic decline, whereas the tighter HRV–HR coupling in long COVID suggests disease-related autonomic dysregulation, potentially linked to impaired flexibility and recovery underlying post-exertional malaise (PEM).

The pathophysiology of autonomic nervous system dysfunction in long COVID is unclear, but some recent studies provide some insights. The autonomic modulation of the (para)sympathetic activity during exercise is partly mediated by the release of catecholamines. Autoantibodies against various catecholamine receptors involved in the autonomic nervous system have been observed in patients with long COVID and these correlate with symptom severity [31]. Whether autonomic nervous system dysfunction is a response to a viral infection for brain stem function [32], viral persistence, other neuropathophysiological alterations [33] or autoimmunity [34] is currently not clear. Also, how acute exercise modulates the response of the immune system to the viral persistence or autoantigen presentation, and how this links to the onset of post-exertional malaise in patients with long COVID is currently unknown.

### Recovery After Moderate to Heavy Exercise

As the parasympathetic nervous system is more active during lower-intensity exercise, the first ventilatory threshold has been described as a point where sympathetic activation begins to increase, as reflected in HRV dynamics [35]. Recovery of parasympathetic activity is typically delayed (up to 60–120 min) after exercise above VT1, even in highly-trained athletes [35]. In our real-life study, we observed that in healthy individuals, HRV values recover within a few hours after the completion of exercise, as the ratio of sympathetic and parasympathetic activities shift back towards mainly parasympathetic activities, which aids in recovery. However, this increase in HRV during the recovery of exercise was delayed for a significantly longer period in patients with long COVID and did not return to the same values as in healthy controls, especially after exercise performed above VT1 and most notably in the moderately impaired group. This blunted HRV recovery after exercise suggests an insufficient or delayed response in the reduction in sympathetic nervous system activity, and/or an increase in the parasympathetic activity after exercise. These inabilities to return heart rate variability back to baseline values indicate that exercise recovery is impaired in patients with long COVID, particularly in those with moderate impairment, characterized by a VT1 below 15 ml O_2_/min/kg. This could contribute to prolonged physiological stress and a delayed recovery period. Above the VT1, the sympathetic activity and stress hormone concentrations (i.e. cortisol and catecholamine) typically increase [36], but we did not measure stress hormone concentrations and time courses during and after high-intensity exercise in patients with long COVID.

Additionally, we observed lower night-time HRV in patients with long COVID following day-time exercise of long duration and/or moderate to high intensity. This suggests that moderate-to-intense physical activity may continue to suppress parasympathetic activity during subsequent sleep, potentially resulting in reduced recovery.

Our findings, using real-life data from patients, provide further insights into the pathophysiology of post-exertional malaise (PEM), in which exertion leads to worsening of symptoms and longer recovery times [1]. We do not know which processes underlie the induction of PEM after physical exercise. Since PEM symptoms are not limited to skeletal muscle-associated abnormalities [1], we provide evidence that acute exercise leaves an autonomic nervous system signature that could, at least in theory, initiate multi-organ symptomology including unrefreshing sleep and worsening of other symptoms. The underlying pathophysiology of PEM and the link with abnormal autonomic nervous system activities is unknown, as we do not have recordings of PEM episodes in this real-life study. As such, more controlled studies need to be performed to provide a deepened mechanistic understanding of the role of the autonomic nervous system in PEM and the pathology of long COVID.

Currently, no curative treatment for PEM is available, and patients are advised to limit exercise in the risk zone for PEM induction. This risk zone for PEM induction is currently unknown, but as HRV recovery responses were particularly disrupted with exercise of a longer duration (> 60 min) and at intensities near VT1, we hypothesize that a process occurring around VT1 is the initiator of PEM, and that as such, a heart rate above VT1 can be used as a surrogate measure for an elevated risk for PEM induction. Anecdotal evidence from patients with long COVID suggests that this risk for PEM induction is time- and patient-dependent, and is not only caused by muscular activities, but also by cognitive and mental exertion. As VT1 is mechanistically related to altered peripheral lactate production, this should be interpreted as a practical, surrogate marker for the risk zone for PEM induction. VT1 is also associated with increased sympathetic activity and release of cortisol and catecholamines (cortisol and (nor)adrenaline) into the blood stream [36], but it is unknown whether the increase in sympathetic activity or the circulating catecholamines, or other hormonal alterations provide a more mechanistic underpinning of PEM induction. Whether an abnormal physiological stress response or disturbed relaxation response underlies the acute onset of PEM deserves further study.

### Clinical Implications

#### Biomedical Basis for Long COVID

Our findings provide further evidence of physiological alterations during and after physical exercise in patients with long COVID that are distinct from healthy controls. Autonomic dysfunction and impaired recovery in HRV in patients with long COVID contribute to our understanding of abnormal exercise responses in long COVID, and likely also patients with post-acute infectious syndrome (PAIS) or myalgic encephalomyelitis/chronic fatigue syndrome (ME/CFS) who also suffer from PEM.

#### Application of Wearables

Wearable devices can be used for monitoring HRV over multiple days in both patients and controls during sleep, daily life activities and post-exercise recovery. These devices can be used both in clinical practice and by patients themselves (preferably guided by experts) to identify stressful moments, overexertion and recognize personalized patterns that impact recovery. By utilizing this data, clinicians can offer more tailored advice on pacing and exercise, ensuring that patients avoid overexertion and exacerbation of symptoms. This approach also empowers patients by providing real-time insights into their physical and recovery states, helping them make informed decisions about their activities and avoid actions that could lead to worsening symptoms.

#### Guidelines for Exercise Intensities

The impaired HRV recovery seen in patients with long COVID emphasises the importance of managing exercise intensities and duration carefully, as overexertion not only impacts immediate recovery, but can also disrupt sleep and subsequent recovery. Our results indicate that physical exercise with increasing intensity and duration significantly reduces subsequent HRV during the night, impairing heart rate variability recovery during sleep. Sports physicians can conduct a submaximal exercise test to determine the heart rate at VT1, which helps patients in providing a usable marker for the risk zone for PEM induction during physical exercise. We consider VT1 to represent a surrogate marker for the post-exertional malaise (PEM) risk zone, which is likely associated with a physiological process undergoing change around VT1, such as sympathetic nervous system activation, hormonal fluctuations or the release of stress-related mediators. Cognitive or mental exertion may trigger a similar physiological alteration as during physical exertion above VT1. Thus, VT1 should be interpreted as a practical and measurable surrogate for the underlying biological risk zone for PEM induction. Continuous heart rate and HRV monitoring throughout the day can be done using wearables, which help improve recovery and mitigate symptoms of post-exertional malaise.

### Limitations

This real-life study using HRV monitors in patients has several limitations. Firstly, the real-life setting of this study meant that a strict experimental protocol could not be applied. A wide range of (combined) daily life activities were recorded in the logbook, which increased subject-to-subject variability. Some activities were reported by only a small number of participants, limiting the statistical power. Current best treatment for patients with long COVID include the avoidance of PEM-inducing exercise, and as such, we have not induced PEM in a controlled manner in this study. The goal of heart rate pacing below VT1 was to prevent PEM. Without heart rate pacing, the exertion in the long COVID group might have been higher, leading to even more pronounced differences compared with healthy controls. In this retrospective field study, we could not control the timing of the exercise during the day, and the nature of the post-exercise period. Nonetheless, regardless of the inherent variability in the post-exercise time period, we observed longitudinal and cross-sectional differences in the HRV recovery profiles. Future prospective and laboratory-controlled studies are required to validate these findings. We acknowledge that orthostatic tachycardia is a common issue in long COVID and could influence heart rate measurements, particularly during upright activities. Lastly, we lacked information on participants’ pre-disease exercise capacity and their COVID-related burden on quality of life, making it difficult to disentangle true disease-related impairment from pre-existing fitness levels, particularly in patients with mild and moderate impairment. Symptom questionnaires would have allowed us to correlate abnormal HRV recovery after exercise with PEM symptomology. The severity and time since the initial COVID-19 infection in long COVID participants was unknown, which may have influenced HRV values. We acknowledge the relatively small control group with a completed exercise test. These potential limitations should be accounted for when interpreting heart rate variability in future research.

## Conclusions

In this study, we provide evidence that heart rate variability is lower during daily life activities in patients with long COVID and remains lower for longer after exercise. The continuous measurement of HRV provides an easy-to-use non-invasive method that may help limit physical overexertion and prevent induction of PEM in patients with long COVID. HRV can be easily measured using wearables during daily life activities in patients, and can also be used for future clinical intervention therapies on treating or reducing PEM in patients.

## Supplementary Information

Below is the link to the electronic supplementary material.

Supplementary file1 (DOCX 5149 kb)

## Data Availability

The pseudonymised data is available upon reasonable request from the corresponding author.

## Declarations

### Equity, diversity and inclusion

This study included both men and women of diverse age and cultural, educational and socioeconomic backgrounds. Diversity was not specifically targeted in recruitment.
