# Supplementary material for: Wearable Heart Rate Variability Monitoring, Autonomic Dysfunction and Post-exertional Malaise in Long COVID: An Observational Study
Source: Sports Med. 2026 Jul 25;56(12):3141–53. doi: 10.1007/s40279-026-02487-4 (PMC13759688; doi:10.1007/s40279-026-02487-4)
Supplement: Supplementary file 1 [file 40279_2026_2487_MOESM1_ESM.docx]

*Supplemental file related to:*

**Wearable heart rate variability monitoring, autonomic dysfunction and post-exertional malaise in Long COVID: an observational study**

Twan Ruijgt ^1^, Anouk Slaghekke ^1^, Anneke Ellens ^2^, Kasper W. Janssen ^2^*, Rob C.I. Wüst ^1^*

*^1^ Department of Human Movement Sciences, Faculty of Behavioural and Movement Sciences, Amsterdam Movement Sciences, Vrije Universiteit, Amsterdam, The Netherlands.*

*^2^ DeSportarts, department of sports and exercise medicine, Utrecht, The Netherlands.*

**: Correspondence: Kasper Janssen PhD, sports and exercise medicine clinic, DeSportarts, Dublinstraat 2, 3541 CD Utrecht, The Netherlands, email address: kasperjanssen@desportarts.nl, and Rob CI Wüst PhD, Department of Human Movement Sciences, Faculty of Behavioural and Movement Sciences, Vrije Universiteit Amsterdam, Van der Boechorststraat 7, 1081 BT Amsterdam, The Netherlands. E-mail address: r.wust@vu.nl.*

**Supplemental tables and figures**

**Supplemental Table 1.** Heart rate (HR) during daily life activities. Median [interquartile range] (number of participants).

|  | **Healthy controls** | **Long COVID** | *P* value |
| --- | --- | --- | --- |
|  | HR (beats.min^-1^) | HR (beats.min^-1^) |  |
| Sleep | 55.4 [52.7-64.0] (21) * | 63.8 [59.1-70.5] (119) * | *0.002* |
| House chores | 86.4 [79.4-95.9] (18) | 89.1 [81.8-95.4] (108) | 0.413 |
| Eating | 74.9 [70.2-84.9] (20) | 82.4 [77.9-90.0] (105) | 0.076 |
| Working | 76.1 [69.0-83.0] (20) | 78.8 (72.5-86.4) (82) | 0.930 |
| Traveling | 78.6 [73.7-85.2] (14) | 83.6 [78.0-9.2] (86) | 0.142 |
| Relaxing | 73.2 [62.4-79.1] (16) | 74.4 [68.4-83.9] (100) | 0.168 |
| Watching TV | 71.1 [64.6-76.4] (14) | 74.4 [69.1-79.9] (99) | 0.201 |
| Walking | 101.3 [82.5-103.6] (13) | 91.7 [83.8-100.1] (97) | 0.317 |
| Exercising | 114.6 [108.1-119.8] (21) * | 102.7 [92.6-111.9] (103)* | *<0.001* |
| Getting ready | 82.7 [70.3-89.8] (7) | 86.9 [80.5-93.8] (74) | 0.439 |
| Relaxing exercise | 58.4 [58.4-58.4] (1) | 78.9 [72.3-89.3] (58) | 0.273 |
| Reading | 73.9 [65.2-76.4] (7) | 74.1 [67.8-83.8] (60) | 0.721 |
| Remaining activities | 76.7 [71.2-85.0] (21) * | 83.8 [77.0-96.2] (110) * | *0.019* |

**Supplemental Table 2.** Heart rate variability (HRV) during daily life activities. Median [interquartile range] (number of participants).

|  | **Healthy controls** | **Long COVID** | *P* value |
| --- | --- | --- | --- |
|  | HRV (rMSSD) | HRV (rMSSD) |  |
| Sleep | 45.2 [28.6-61.0] (21) * | 32.6 [24.3-46.9] (119) * | *0.027* |
| House chores | 23.6 [17.6-34.6] (18) | 20.9 [15.1-26.6] (108) | 0.140 |
| Eating | 29.4 [19.3-41.7] (20) * | 22.5 [17.2-29.8] (110) * | *0.005* |
| Working | 31.2 [20.0-37.4] (20) | 25.6 [20.3-33.2] (83) | 0.239 |
| Traveling | 29.6 [20.6-37.5] (14)* | 22.4 [16.0-28.4] (99)* | *0.007* |
| Relaxing | 35.5 [16.9-47.4] (16) | 27.8 [19.2-39.0] (108) | 0.240 |
| Watching TV | 26.7 [19.1-43.5] (14) | 25.1 [20.0-35.3] (106) | 0.365 |
| Walking | 17.0 [11.5-23.7] (13) | 15.6 [10.9-20.5] (102) | 0.593 |
| Exercising | 12.4 [8.9-17.1] (21) | 13.4 [10.5-18.0] (113) | 0.253 |
| Getting ready | 24.0 [17.1-34.4] (7) | 23.8 [16.3-29.5] (57) | 0.639 |
| Relaxing exercise | 37.8 (1) | 26.1 [20.3-40.3] (54) | 0.179 |
| Reading | 20.3 [14.5-45.4] (7) | 26.2 [19.6-33.7] (73) | 0.647 |
| Remaining activities | 32.2 [19.7-43.5] (21) * | 24.2 [18.8-32.8] (120) * | *0.016* |

**Supplemental Figure 1.** (**A**) HRV was negatively associated to age in both patients with long Covid (p=0.0002, R^2^=0.11) and healthy controls (p=0.0011, R^2^=0.44). (**B**) Within the group of patients with long COVID, HRV was significantly lower in females (p=0.0243). Therefore, HRV statistical tests were corrected for age and sex.

**Supplemental Figure 2.** Heart rate variability (HRV) significantly correlated to heart rate (HR) in both long COVID (p<0.0001, R^2^=0.34) and healthy controls (p=0.042, R^2^=0.20).

**Supplemental Figure 3: Subgroup analysis based on same group size (see supplementary** **table 3).** A) Longitudinal trace of Heart Rate Variability over a 24-hour period following moderate exercise (90-100% VT1) in controls and matched Long COVID patients. Overall, recovery trajectories still differed significantly between groups. Furthermore, controls showed a significant increase in HRV compared to exercise baseline by 5 hours post-exercise (indicated by the blue circle). In contrast, the Long COVID group showed a delayed response, reaching this threshold only at 10 hours post-exercise. B)

**Supplemental Table 3.** **Subgroup analysis based on same group size (n=21)** with age, sex, and BMI matched patients and healthy controls.

|  | ***Controls***  ***(n=21)*** | ***Long COVID***  ***(n=21)*** | ***p-value*** |
| --- | --- | --- | --- |
| ***Age (Years)*** | *42.3 +/- 12.8* | *42.1 +/- 12.1* | *0.97* |
| ***Sex (Male)*** | *10* | *10* | *>0.99* |
| ***BMI (kg/m^2^)*** | *23.4 +/- 2.6* | *23.7 +/- 2.5* | *0.75* |
